# Elastic Property Evaluation of Fiberglass and Epoxy Resin Composite Material Using Digital Image Correlation

**DOI:** 10.3390/ma17153726

**Published:** 2024-07-27

**Authors:** Dalferson Yoras, Sylwia Makowska, Agnė Kairytė, Jurga Šeputytė-Jucikė, Luis Roberto Centeno Drehmmer, Maikson Luiz Passaia Tonatto

**Affiliations:** 1 Group on Mechanics of Materials and Structures, Campus Cachoeira do Sul, Federal University of Santa Maria, Cachoeira do Sul 96503-205, Brazil; dalfersonyoras123@hotmail.com (D.Y.); luis.drehmer@ufsm.br (L.R.C.D.); maikson.tonatto@ufsm.br (M.L.P.T.); 2 Institute of Polymer and Dye Technology, Faculty of Chemistry, Lodz University of Technology, Stefanowskiego 12/16, 90-924 Lodz, Poland; sylwia.czlonka@edu.p.lodz.pl; 3 Laboratory of Thermal Insulating Materials and Acoustics, Institute of Building Materials, Faculty of Civil Engineering, Vilnius Gediminas Technical University, Linkmenų St. 28, 08217 Vilnius, Lithuania; jurga.seputyte-jucike@vilniustech.lt

**Keywords:** composite materials, strain measurements, epoxy resin, fiberglass, manual lamination, mechanical properties

## Abstract

This study focused on evaluating the sensitivity and limitations of the simplified equipment used in the Digital Image Correlation (DIC) technique, comparing them with the analog extensometer, based on the mechanical property data of a composite made of fiberglass and epoxy resin. The objectives included establishing a methodology based on the literature, fabricating samples through manual lamination, conducting mechanical tests according to the ASTM D3039 and D3518 standards, comparing DIC with the analog extensometer of the testing machine, and contrasting the experimental results with classical laminate theory. Three composite plates with specific stacking sequences (${\left[0\right]}_{3}$, ${\left[90\right]}_{4}$, and ${\left[\pm 45\right]}_{3}$) were fabricated, and samples were extracted for testing to determine tensile strength, modulus of elasticity, and other properties. DIC was used to capture deformation fields during testing. Comparisons between data obtained from the analog extensometer and DIC revealed differences of 11.1% for the longitudinal modulus of elasticity $E_{1}$ and 5.6% for $E_{2}$. Under low deformation conditions, DIC showed lower efficiency due to equipment limitations. Finally, a theoretical analysis based on classical laminate theory, conducted using a Python script, estimated the longitudinal modulus of elasticity $E_{x}$ and the shear strength of the ${\left[\pm 45\right]}_{3}$ laminate, highlighting a relative difference of 31.2% between the theoretical value of 7136 MPa and the experimental value of 5208 MPa for $E_{x}$.

## 1. Introduction

Composite materials highlight their significant structural efficiency, demonstrating a strong relationship between high strength and low weight [1,2]. These high-strength polymeric materials have the potential to replace metals and can constitute up to 50% of the total mass of aircraft. The use of composites in aircraft manufacturing offers several advantages, including weight reduction, improved structural efficiency, corrosion resistance, and adaptable design, as well as reduced vibrations and noise, as noted by [3]. Research in composite materials is crucial for advancing innovative technologies and addressing contemporary challenges. Baggio et al. [4] emphasizes the importance of understanding the characteristics and behavior of these materials for their proper configuration in terms of sizing, construction, and application.

Strain measurements are essential for obtaining precision in elastic properties, promoting a deeper understanding of the mechanical behavior of these materials. The heterogeneity of composite materials [5,6] makes the strain measurements, whose properties are influenced not only by the material composition but also by the geometric design of the structural elements, particularly difficult.

From a theoretical perspective, analytical theories can estimate the elastic constants of laminates. Macromechanical behavior refers to the average macroscopic mechanical properties of laminates, which can be analytically evaluated from a theoretical perspective. Researchers, such as Belaid et al. [7], indicate that these properties can be obtained through experimental tests or calculated based on micromechanics. This allows for the estimation of laminate behavior under various load combinations. According to the classical laminate theory, as discussed by Kaw [8], theoretical data on global and local stresses, strains, and potential failures can be obtained. This theory assumes an elastic–linear behavior for composite materials, a characteristic that has been verified in E-glass/epoxy laminates. Neto [9] highlights that composite materials exhibit a greater capability of following a linear stress–strain relationship when subjected to loading compared to metallic materials, making it favorable to consider them as linear elastic materials.

From an experimental perspective, Digital Image Correlation (DIC) techniques are widely employed for this purpose, offering precise analysis of displacements and strains. This capability is essential for characterizing and optimizing composite materials, which are extensively used in industries such as aerospace, military, automotive, and sports. DIC works by capturing the displacement vector of each pixel point on the sample surface through a comparison of its position in images taken before loading and after deformation. The accuracy of this process heavily relies on the presence of a unique and random pattern of markers, as emphasized by Sabik et al. [10].

An NBC News report [11] with experts discussing the implosion of the submersible emphasizes the need for numerous specific tests and analyses for this type of material. The analysis of correlation between the initial and final images allows for determining displacement fields, from which sample deformations are derived. The DIC-2D technique requires only a high-definition camera positioned orthogonally to the surface of the test object, maintaining a fixed distance from the camera to the sample, as emphasized by [12,13]. Calibration is based on comparing a known measurement with the results obtained by the DIC technique, involving the evaluation of different deformation states to increase the reliability of the generated values. Blenkinsopp et al. [14] demonstrated the importance of establishing precise predefined deformations used as the reference.

Recent studies have explored laminates of composite materials, such as carbon fiber reinforced polymers and thermoplastics, widely used in the aerospace industry. Non-destructive approaches, such as DIC, have been employed to examine the behavior of these materials. Some studies investigated the influence of manufacturing defects and stress concentrations in quasi-static fatigue tests [15]. Others analyzed displacements in steel beams using DIC, dividing the work into experimental setup, image processing, and result comparison [16]. Gonabadi et al. [17] studied the effects of anisotropy in composite structures for renewable energy, such as wind turbine blades, using DIC and microscopy analysis. Feito et al. [18] analyzed carbon fiber reinforced polymer (CFRP) laminates using non-destructive techniques like IRT and DIC to characterize manufacturing defects and stress concentrators during fatigue tests. Hao et al. [19] reported that the DIC technique allowed for full-field displacements and deformations, identifying potential failures.

The study of composite materials, particularly in relation to their mechanical behavior under various conditions, has seen extensive research employing advanced techniques such as DIC. Researchers have investigated aspects like fiber orientation, strain localization, static and fatigue properties, failure modes, and strain distribution using DIC [20,21], thermographic stress analysis [22], Acoustic Emission [23], and conventional mechanical testing [24,25]. This array of studies underscores the versatility and importance of DIC in providing detailed insights into the mechanical behavior of fiber-reinforced composites. However, the sensitivity and limitations of lower-cost equipment in DIC, especially for measuring small strains, are not yet well understood. Identifying the detection limits of strain and displacements for DIC and analog extensometers is crucial for further development in this field.

This study focused on exploring the sensitivity and limitations of DIC techniques and analog extensometers in assessing the elastic properties of fiberglass/epoxy laminates, especially in small strains.

## 2. Materials and Methods

With the aim of developing an assessment of the sensitivity and limits of the equipment used in the DIC technique, the methodology employed for constructing the composite was executed in a standardized manner to minimize potential variations among samples. For the three produced laminated plates, both the fiber and matrix were used in equal proportions. Parameters such as temperature, compaction weight, and curing time were kept constant. Ten samples were taken from each plate, with only the best five samples from each laminate considered for statistical data analysis.

### 2.1. Materials and Manufacturing

The manual lamination process and mechanical tests were conducted in the Mechanical Engineering Laboratory of the Federal University of Santa Maria—Campus Cachoeira do Sul.

**Figure 1:**
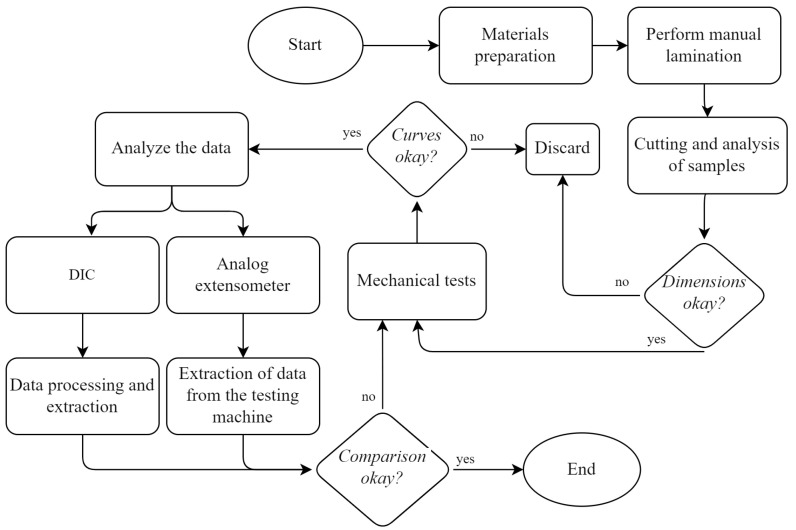
Methodology flowchart.

Figure 1 presents a flowchart describing the main steps that were followed during the conduct of this study, which was planned to be carried out systematically.

In the lamination process, uni-directional E-glass fiber fabric from Texiglass [26] was used in conjunction with fast-curing, low-resistance epoxy resin AR-320, combined with hardener AH-320 from the E-composites brand (E-Composites, Rio de Janeiro, Brasil). To ensure proper compaction of the composite and minimize the formation of air bubbles in the laminate, nylon peel-ply fabric was employed. Additionally, a release agent such as carnauba wax was applied to facilitate the removal of the composite plates after the curing process, along with other materials used in manual lamination.

During the process, three composite plates were fabricated, each with specific dimensions due to variations in sample sizes. Each plate was sized to accommodate a total of ten samples, including tabs. The plates were produced with varying numbers of layers and fiber orientations to meet thickness specifications required by applicable standards for composite materials testing. In the ${\left[0\right]}_{3}$ configuration, three layers of fabric were aligned in the same direction as the applied tensile load. In the ${\left[90\right]}_{4}$ configuration, four layers of fabric were positioned perpendicular to the direction of the tensile load. Finally, in the ${\left[\pm 45\right]}_{3}$ configuration, a total of six layers of fabric were interleaved.

The total curing time for the plates was 36 h, with a controlled temperature of 22 °C in the laboratory, and a compaction weight of 35 kg applied to each plate. Cutting of the plates for sample and tab preparation was conducted according to dimensions specified in standards and detailed in Table 1. A computer numerical control (CNC) machine was utilized for the cutting process, employing water for material cooling to prevent tool clogging.

For the tab bonding process, the same resin used in composite fabrication was employed, necessary only in the ${\left[0\right]}_{3}$ configuration to prevent slippage during testing and reduce sample failures in the machine grip region.

**Figure 2:**
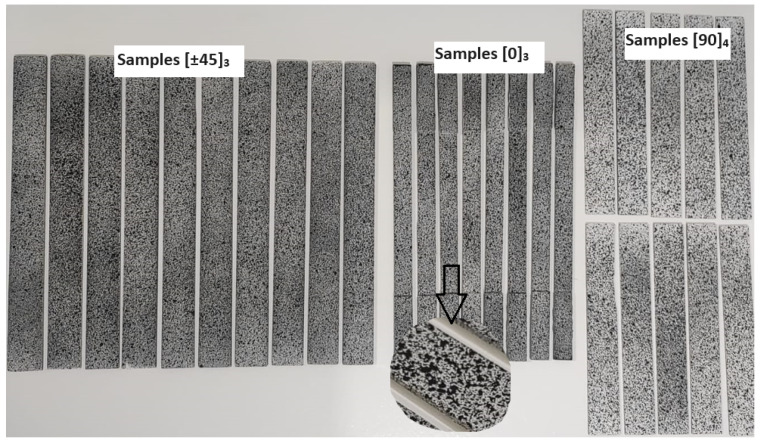
Samples with surface preparation.

Additionally, a final step was required for the samples, which involved surface preparation to enable effective image capture using the DIC technique. This included applying a uniform layer of white paint on all samples, followed by a second coat of black paint applied non-uniformly to create small splatters using a specific and deliberate technique, as shown in Figure 2. This surface preparation was crucial to facilitate the use of the DIC method.

### 2.2. Mechanical Tests

The mechanical tests were conducted following the guidelines established by ASTM D3039 [27] for composite material tensile testing. In the ${\left[0\right]}_{3}$ configuration, stress–strain curves were obtained to determine the modulus of elasticity ($E_{1}$), longitudinal tensile strength ($\sigma_{1}^{T}$), and ($\nu_{12}$). Additionally, tests in the ${\left[90\right]}_{4}$ configuration aimed to determine the modulus of elasticity ($E_{2}$) and longitudinal tensile strength ($\sigma_{2}^{T}$).

Shear testing according to ASTM D3518 [28] was performed on ${\left[\pm 45\right]}_{3}$ samples, allowing for the determination of shear modulus ($G_{12}$), shear strength ($\tau_{12}$), and the elastic modulus ($E_{x}$) of the laminate.

The tests were conducted on a set of thirty samples, evenly distributed with ten samples for each configuration. This procedure resulted in a total of sixty distinct analyses, as each specimen was evaluated using two different methods. Both standards require a minimum of five samples, leading to the selection of the top five performing samples for each configuration. Consequently, the average mechanical properties were calculated exclusively based on these selected five samples.

**Figure 3:**
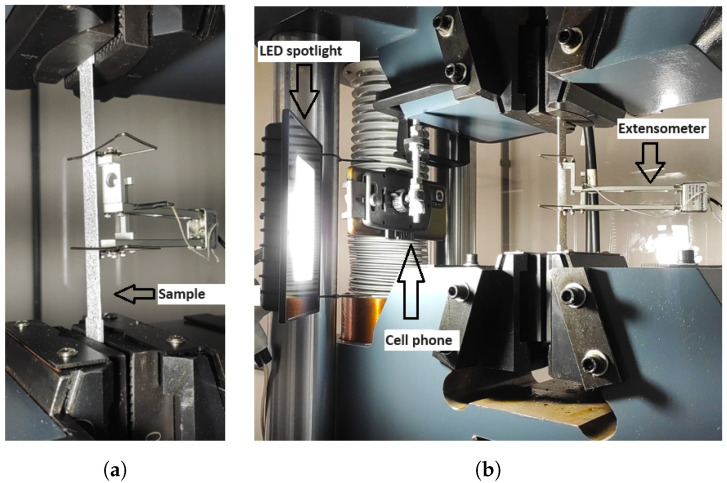
(**a**) Sample clamped in the machine setup. (**b**) During the mechanical test.

The evaluations were carried out using the LEIDA brand universal testing machine, model LW-5000 (Leida equipamentos para laboratórios e ensaios mecânicos, São Paulo, Brasil), with a maximum load capacity of 500 kN, resolution of 0.01 kN, and an approximate error margin of ±1%. The test speed used was 2 (mm/min), as specified by the standard. Figure 3a,b illustrate the configuration of the devices used during the mechanical tests.

The analog extensometer was initially used to record strain data during mechanical tests, as illustrated in Figure 3a. The calculation of composite properties began with determining the modulus of elasticity (*E*) in direction *i*, obtained by the difference in tensile stress (Δσ) divided by the difference in strain (Δϵ), following the standard of 1000 µϵ to 3000 µϵ. The shear modulus was calculated similarly, with deformations from 1500 µϵ to 4000 µϵ.

The tensile strength of the laminates was recorded by the testing machine. The Poisson’s ratio was calculated by the variation in transverse strains relative to longitudinal strains, considering the same initial and final points used in the calculation of the modulus of elasticity.

The dimensions of the test specimens were measured using a 150 mm digital caliper with a resolution of 0.01 mm, following the normative recommendation to measure at least three different points on the sample. During the mechanical tests, images were captured using a strategically positioned smartphone to ensure the best field of view, aided by an LED light for adequate illumination under the sample, as shown in Figure 3b.

Videos were recorded during the tests using a Xiaomi smartphone, model Redmi Note 10 Pro (Xiaomi, Beijing, China), equipped with a 108-megapixel rear camera. This camera was capable of recording videos in 4K resolution, achieving up to 3840 × 2160 pixels and a frame rate of 30 frames per second (FPS). Subsequently, the videos were converted into individual images at 1 s intervals and then imported for analysis into the image processing software to obtain deformation fields. The GOM Correlate 2022 software from Aramis was used for this analysis.

The GOM Correlate software [29] uses the DIC technique. The technique is used to measure the displacements and strains on surfaces by comparing high-resolution images captured before and after deformation. It operates by dividing these images into subsets, identifying unique patterns within each subset, and correlating these patterns between the two images to compute local displacements. Using methods like normalized cross-correlation, DIC calculates displacement vectors for each subset, which are then interpolated to generate full-field displacement maps. From these maps, strains can be derived to analyze the mechanical behavior of materials. This software transforms images into deformation data, allowing comparisons with the analog extensometer. Consequently, it was possible to compile the data into spreadsheets, providing a comprehensive statistical analysis of all tested samples.

The classical laminate theory was used to calculate the engineering constant $E_{x}$ of ${\left[\pm 45\right]}_{3}$ laminate. A script was used in order to automate the resolution of equations, and calculation of stresses, strains, elastic modulus, and failure estimates of the laminate, both on a global and local scale. The mechanical properties for ${\left[0\right]}_{3}$ and ${\left[90\right]}_{4}$ laminates obtained from tests were used as input. This enabled a comparison between the experimental results of the mechanical tests and the theoretical predictions of classical laminate theory for the ${\left[\pm 45\right]}_{3}$ laminate, using global coordinates due to the fiber orientation.

## 3. Results and Discussion

In the initial phase, the results of the ${\left[0\right]}_{3}$, ${\left[90\right]}_{4}$, and ${\left[\pm 45\right]}_{3}$ configurations were presented and discussed using figures, tables, and comparative graphs of the samples. Subsequently, the obtained results from the three tests were analyzed in relation to the relevant literature.

Following the completion of individual tests, a detailed approach was adopted. All collected data were migrated to Excel software, necessitating a meticulous alignment process to correlate the results from the two analysis methods.

An important consideration was the lack of synchronization between the recorded video during the tests and the actual start time of the mechanical test on the machine. To address this, the video recording began a few seconds before the effective start of the mechanical test. This initial time lag received careful attention during the Excel analysis. The purpose of this care was to ensure precise and reliable comparisons of data obtained through these complementary methods, thereby maintaining the integrity and relevance of the final results.

The limit in capturing deformations using the DIC technique with simplified equipment was explored in our study. Despite the challenges posed by simplified setups, valuable insights were gained into the material’s behavior under deformation. A thorough analysis of these limits not only outlined the boundaries for reliable measurements but also underscored the importance of understanding equipment constraints in DIC applications. This understanding enabled a precise interpretation of the experimental results, ensuring that the conclusions were firmly grounded in the capabilities of the technology employed.

The results section is subdivided for each test configuration. The first series covers the ${\left[0\right]}_{3}$ configuration, followed by the ${\left[90\right]}_{4}$ configuration, and finally the ${\left[\pm 45\right]}_{3}$ configuration. These divisions were necessary due to specific adjustments required on the testing machine for each configuration.

### 3.1. Results of Laminate ${\left[0\right]}_{3}$

Initially, greater mechanical strength was observed in the tests because the fibers were aligned with the applied load. However, these specimens also showed low strains, which hindered detailed analysis using the DIC method, largely due to the limited resolution of the equipment used.

An important consideration was made regarding the differences between the methods employed. The primary focus of the tests was on the elastic regime. Therefore, the analog extensometer was removed after reaching approximately 0.7% of strain.

Additionally, during the tests, strains were captured using the DIC method. Since this approach does not require direct contact of the equipment with the sample, it allows for a detailed evaluation of the strain field and the sample’s sensitivity, complementing the information obtained by the analog method. This expands the range of the elastic constants obtained, such as the Poisson’s ratio, for which it is necessary to use two extensometers simultaneously during the test.

**Figure 4:**
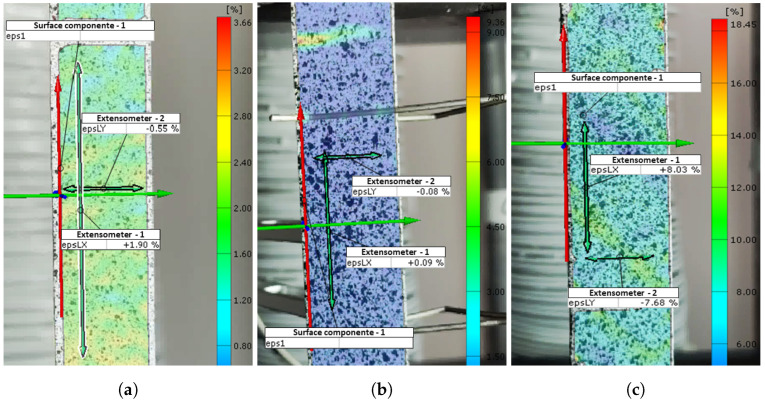
Scheme of longitudinal and transverse digital extensometers for the configurations: (**a**) ${\left[0\right]}_{3}$, (**b**) ${\left[90\right]}_{4}$, and (**c**) ${\left[\pm 45\right]}_{3}$.

Figure 4a displays the strain field along sample 2, representing configuration ${\left[0\right]}_{3}$. The strain field in the fiber direction is observed at a load level of 13.7 kN using DIC. In these representations, digital extensometers are simulated on each specimen to extract strain data.

**Figure 5:**
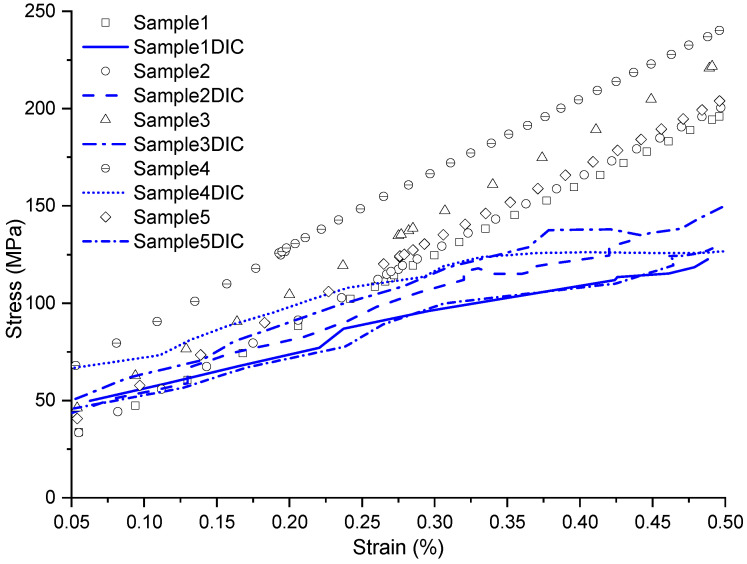
Stress vs. strain graph of the ${\left[0\right]}_{3}$ configuration, within the range where the analyses were conducted.

In the graph in Figure 5, through the stress–strain diagram, ten curves are presented, five of which were captured by the analog extensometer and the other five by the digital extensometer (DIC). Only the strain range required by the standard was demonstrated. A variation between the curves was evident when comparing the strains from the two procedures; however, good agreement was observed, and a trend could be discerned when comparing all curves from the same method.

Based on a statistical study, the five best curves were selected and, from these, the mean, standard deviation, and coefficient of variation were calculated to characterize this laminate. Despite the process variability and experimental measurement, the larger number of samples tested and the selection of the best ones reduced the uncertainties in the results.

It was found that the curves obtained with the analog extensometer exhibited greater uniformity than those obtained with DIC. This could be explained by two reasons: first, the analog method collected data at intervals of 0.1 s, while the digital method did so every 1 s, resulting in fewer points to plot the graph, affecting the linearity of the curves. The second point to consider is that this configuration involved low strains, making it difficult for the camera to capture the deformation in the same proportion as the analog extensometer, showing a certain limitation of the equipment, such as the camera. In other words, the DIC captured, for example, a variation of about 0.3% strain in a shorter time interval than the analog extensometer, showing that it has lower sensitivity to low deformations. Therefore, when comparing the curves for the same specimen at the same strain point, the DIC curve presented lower stress.

Table 2 presents the results of the elastic moduli, one obtained through the analog extensometer and the other with the digital extensometer using the DIC technique. Additionally, the tensile strength values in the fiber direction were described, obtained through the load cell of the testing machine, relating the applied force to the cross-sectional area of the sample. Furthermore, Poisson’s ratio in the 12-plane was included.

**Figure 6:**
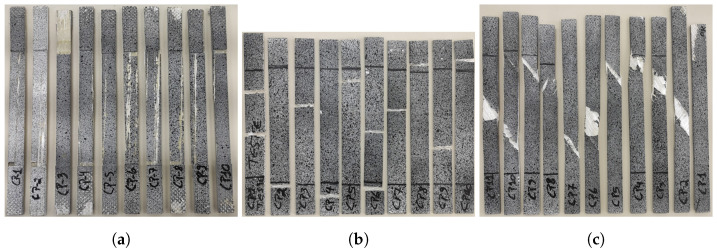
Samples after mechanical tests: (**a**) ${\left[0\right]}_{3}$, (**b**) ${\left[90\right]}_{4}$, and (**c**) ${\left[\pm 45\right]}_{3}$.

Figure 6a shows all the test specimens from the ${\left[0\right]}_{3}$ configuration that were subjected to testing. Among them, some specimens were discarded due to slippage in the fixation elements (tabs), which compromised the data analysis. In the remaining specimens, as mentioned earlier, failure occurred consistently, following the longitudinal direction of the fibers.

In this configuration, a significant complication was noted in capturing strains due to the alignment of the laminate reinforcement in the same direction as the applied load. This alignment resulted in a stiffer laminate, which made the precise detection of strains challenging.

### 3.2. Results of Laminate ${\left[90\right]}_{4}$

The specimens from the ${\left[90\right]}_{4}$ configuration are depicted in Figure 6b after the tensile tests were conducted. This configuration encountered difficulties regarding the coherence and location of failures in the samples. In some cases, failure occurred within the grip, while, in others, it occurred in the center of the sample.

It was observed that all failures in the specimens occurred in the fiber direction, regardless of where they manifested, considering the displacement speed of the universal testing machine, which was 2 mm/min.

The tests revealed excessively low loads compared to other conditions, due to the transverse alignment of the fibers relative to the applied load. Additionally, the high variability observed in the readings of the analog extensometer was a consequence of the sample’s fragility, where small variations in load resulted in significant changes in extensometer readings, making it challenging to obtain consistent and reliable data, particularly due to the high robustness of the machine used relative to the load level.

When comparing this configuration with the previous ${\left[0\right]}_{3}$, the presence of anisotropy in the composites was noted. Considering it is the same material, the only difference lies in the direction in which the load is applied relative to the fibers, resulting in distinct properties. Moreover, the fact that the ${\left[90\right]}_{4}$ configuration has an additional reinforcement layer compared to ${\left[0\right]}_{3}$ suggests that, if both configurations had the same number of reinforcement layers, the discrepancy between the presented values could be even greater.

Regarding the strains obtained by DIC, Figure 4b displays the strain field in the test for a load level of 395 N. It was evident that there was reduced strain in both the longitudinal and transverse extensometers. In this configuration, the camera encountered difficulties capturing strains due to the high sensitivity of the sample, low strains, and limited testing time, resulting in a shorter data recording period compared to other laminates and restricting the detailed analysis of the sample’s behavior over time.

**Figure 7:**
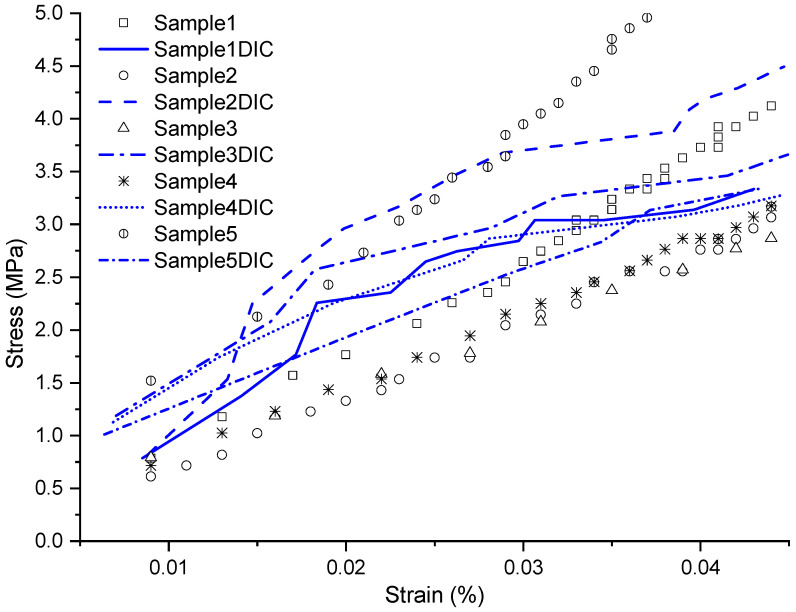
Stress vs. strain graph of the ${\left[90\right]}_{4}$ configuration, within the range where the analyses were conducted.

In the stress–strain diagram of Figure 7, ten curves representing the laminate are presented, with five captured by the analog extensometer and the other five by the digital extensometer from DIC.

Table 2 presented the results of the elastic moduli, one obtained through the analog extensometer and the other with the digital extensometer using the DIC technique. Additionally, the tensile strength values in the transverse direction to the fibers were described, obtained through the load cell of the testing machine, relating the applied force to the area of the sample’s cross-section.

In this configuration, a difficulty was encountered due to the high sensitivity of the samples, where the alignment of the fibers transverse to the load amplified this effect, requiring precise detailing within a short testing period.

### 3.3. Results of Laminate ${\left[\pm 45\right]}_{3}$

Figure 6c presented the third series of tests on the machine, where all samples failed in a coherent and consistent manner, following the direction of the fibers and accompanied by a reduction in the cross-sectional area, something that was not observed in other configurations. The test naturally demonstrated the highest strain due to the alignment of the fibers at a 45-degree angle to the applied load. Additionally, it was confirmed that these specimens exhibited good shear strength.

During the execution of this test, it is important to highlight that the analog extensometer was used until the deformation reached approximately 1%, as a safety measure to prevent equipment damage in case of unexpected sample failure. The displacement speed of the universal testing machine was 2 mm/min.

For the strains obtained by DIC, as demonstrated in Figure 4c, the strains recorded in both the longitudinal and transverse extensometers were substantially larger compared to previous configurations. This observation makes this laminate particularly interesting for study.

The greater magnitude of strains provides significant advantages in characterizing laminated composite materials. This approach allows for a more robust and detailed data collection, as well as enabling the analysis of the initiation and propagation of progressive damage, which are crucial elements in understanding the behavior of these materials under load. The higher strains recorded by strain gauges provides a broader margin for interpreting the results, facilitating the identification of trends and specific behaviors of the laminate.

The deformation capability of each laminate is inherently related to its stacking sequence, which is essential for distinguishing one laminate configuration from another and for evaluating the material’s strength in progressive damage scenarios. The focus of this study lies in analyzing low deformations, despite the laminates being capable of exhibiting higher deformations.

The present study corroborates the findings of Xu et al. [15], as the differences between the deformation obtained by DIC and other methods, such as analytical and numerical, are greater for cases with lower levels of deformation. The analysis of deformations in hybrid composites reinforced with natural fibers, conducted by Xu et al., revealed important insights into the mechanical behavior of these materials under load. Longitudinal and transverse deformations were obtained using DIC for flax/epoxy, jute/epoxy, flax/glass/epoxy, and jute/glass/epoxy composites. The results showed that the addition of glass layers reduced the transverse contraction coefficients compared to composites reinforced only with natural fibers, increasing the difference between the methods. This correlates with the present study, as for the 0 and 90 samples with lower levels of deformation, the difference was greater when compared to the 45 samples.

**Figure 8:**
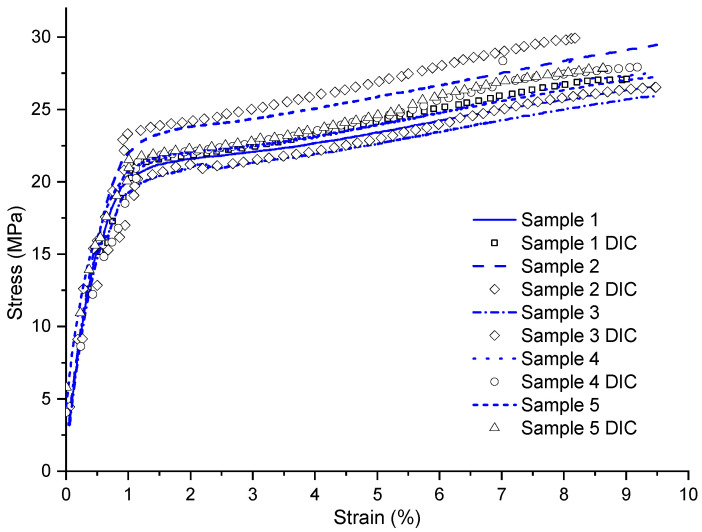
Stress vs. strain graph of the ${\left[\pm 45\right]}_{3}$ configuration with strain obtained by DIC and analog extensometer.

In Figure 8, ten curves are represented, enabling a comprehensive comparison between the accuracy of DIC and the analog extensometer. Both approaches revealed notable fidelity in capturing strains, given that this configuration had a greater strain than the other conditions, thus facilitating the acquisition of deformation fields. It is observed that the curves obtained with DIC have small markers on their respective curves to reduce visual clutter in the diagram.

**Figure 9:**
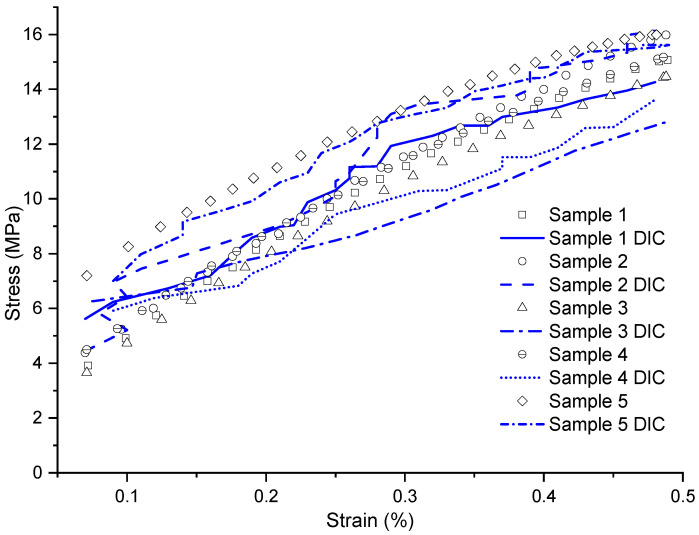
Stress vs. strain graph of the ${\left[\pm 45\right]}_{3}$ configuration, within the range where the analyses were conducted.

In Figure 9, using the stress–strain diagram, ten curves are presented to represent the laminate, employing both the analog extensometer method and the digital DIC extensometer method. All curves start with similar stress levels. However, the curves obtained with the analog extensometer remain stable, whereas those obtained with the digital extensometer show slight variations. This is because the analog extensometer recorded data every 0.1 s, whereas the digital method did so every 1 s.

In this specific configuration, the camera used demonstrated a remarkable ability to capture deformations extremely accurately, aligning closely with the data recorded by the analog extensometer. This precision can be mainly attributed to the fact that this specific configuration exhibits higher levels of strain compared to the other conditions. Consequently, the data recording period was relatively longer compared to the other laminates, allowing for a more detailed and precise analysis of the sample’s behavior over time.

In Table 2, another data point presented is the shear modulus in the 12 plane, which can only be obtained with the assistance of an additional digital extensometer in the transverse direction, requiring the simultaneous use of two extensometers attached to the sample. The values of tensile strength in the longitudinal direction of the laminate are described, as well as the shear strength in the 12 plane, which essentially equals half of that value, both obtained through the testing machine, relating the applied force to the cross-sectional area of the sample. Overall, the calculated coefficient of variation was small, indicating low data dispersion, and thus the number of samples was adequate.

### 3.4. Discussions of Results

The comparison of the results obtained with the literature in the field was conducted using the data compiled in Table 2. Mechanical properties such as the elastic modulus in directions 1 and 2, shear modulus in the 12 plane, and Poisson’s ratio in planes 12 and 21, as well as tensile strength in directions 1 and 2 and shear strength in the plane, were collected based on the average values obtained from a rigorous statistical analysis of the tests, both by the DIC method and the analog method. Additionally, comparisons with the literature were provided to validate the results obtained in the tests.

All cited studies used unidirectional E-glass fabric. In study [31], polyester resin was used, which could be disregarded for comparison with the elastic modulus in the fiber direction, as the reinforcement configuration is the most critical aspect. On the other hand, the studies [32,33] used epoxy resin similar to that tested experimentally in the present study, but from different manufacturers. Other properties were obtained using Mech-G software [30] developed by the Composite and Nanocomposite Materials Group (GCOMP) at LAPOL/UFRGS.

It is important to note that, in all studies, the tensile tests followed the ASTM D3039 standard, ensuring the comparability of the results. Another fundamental point discussed in the studies was the sample manufacturing method, which was mostly carried out through hand lay-up, except for the study by Reis, which employed the vacuum infusion process.

It is worth emphasizing that the manufacturing process of composite materials significantly influences the magnitude of the properties obtained experimentally. The study [34] highlighted that the manufacturing technique used can significantly affect the mechanical properties of composites, including tensile strength and elastic modulus.

In laminate ${\left[0\right]}_{3}$, the stress–strain curves obtained by the analog extensometer showed an average modulus of elasticity of 38,538 MPa. Conversely, the curves obtained by the digital extensometer exhibited some variations, resulting in an average modulus of elasticity value of 34,459 MPa. This represented a relative difference of 11.1%; in the literature, values obtained in similar studies also varied for this same property.

The Poisson’s ratio 12 found was 0.29, very close to the value of 0.28 obtained in the [30] reference. The greatest discrepancy between the results of this test and those from the literature was in the tensile strength in the fiber direction, which was 1035 MPa, contrasting with the 567.4 MPa obtained in the mechanical tests. In the cited experimental studies, the tensile strength limits were 288.8 MPa, 410 MPa, and 445.4 MPa, for [31,32,33], respectively. These values were more consistent with the magnitude obtained in the tests, suggesting that the number of plies and the manual lamination process interfere with the results obtained.

In laminate ${\left[90\right]}_{4}$, DIC showed difficulties in capturing strains due to the high sensitivity of the samples. However, it is noteworthy that the obtained data significantly approached the values found in the literature. The results were, respectively, 9581 MPa using DIC, 10,152 MPa with the analog method, and 12,411 MPa in the literature, using [30] as a reference.

Regarding tensile strength in the direction transverse to the fibers, the values were 7.5 MPa obtained in the tests and 48 MPa in the literature. However, comparing the strength obtained in the tests with the epoxy resin strength indicated by the manufacturer [35], which is 7.5 MPa, coherence becomes evident. This is because, in this test, the properties most significantly requested are those of the resin, and the fibers hardly influence the strength in the transverse direction.

In laminate ${\left[\pm 45\right]}_{3}$, comparing the shear modulus properties obtained with the mechanical tests, they were respectively 2058 MPa and 5515 MPa obtained with the Mech-G software, while, in study [33], a value close to 2270 MPa was obtained. Regarding shear strength in plane 12 of the tests of this study, 28.2 MPa was obtained, while, in the literature, it was 68 MPa and 31.4 MPa, for [30,33], respectively.

The values of the data obtained in the tests differed from the indicated studies, mainly due to the number of reinforcement layers used in the composite. Another relevant factor was the type and characteristics of the manufacturing process, including the curing methods of the laminate. Since the data were experimentally obtained, there are unquantified uncertainties that influence the final result. This disparity was understandable, as the materials, despite being the same (unidirectional E-glass fiber and epoxy resin), are from different manufacturers and undergo different processes and treatments, which consequently result in different characteristics and properties obtained for the laminate.

Moreover, the analytical calculation based on classical laminate theory was used to obtain the engineering constants of laminates, including the longitudinal elastic modulus $E_{x}$. The data used to feed the method were primarily extracted from Table 2, pertaining to laminates ${\left[0\right]}_{3}$, ${\left[90\right]}_{4}$, and ${\left[\pm 45\right]}_{3}$, the latter considering data obtained from local coordinates. Additionally, information such as stacking sequence, average laminate thickness, and number of layers were also incorporated.

Table 3 presents the average values of the results obtained for the engineering constant of the ${\left[\pm 45\right]}_{3}$ laminate, using both the DIC and analog methods, considering the global coordinates of the system.

Regarding the modulus of elasticity obtained for this laminate, the results were consistent as analyzed through the classical laminate theory. It is important to note that the analysis was conducted without considering interactions related to progressive damages occurring during the failure of composite materials. In other words, the approach adopted was idealized, resulting in a modulus of elasticity value of 7136 MPa. Conversely, the experimentally obtained value for the modulus of elasticity was 5208 MPa. This discrepancy represents a relative difference of 31.2%, primarily attributed to the manufacturing process involving unquantified uncertainties combined with progressive damage during material failure in the experimental tests.

## 4. Conclusions

This study investigated the mechanical properties of three composites with different stacking sequences reinforced with E-glass fibers, using DIC measurements and an analog extensometer during tensile tests. The DIC method, performed with simplified equipment, effectively determined the elastic and shear moduli as well as the Poisson’s ratio of the analyzed laminates. Additionally, the strain fields obtained by the DIC technique contribute to a better understanding of sensitivity and limitation of the simplified equipment.

Visual analysis of the specimens after testing revealed that failures predominantly occurred at the interface between the fiber and the composite matrix, without rupture of the glass fiber filaments. This failure mechanism was consistent across all examined cases. In the ${\left[0\right]}_{3}$ laminate, the samples exhibited high mechanical strength, averaging 567.4 MPa, and low strain, with the limited resolution of the equipment detecting minimal strains. In the ${\left[90\right]}_{4}$ laminate, lower strains and tensile strength were observed, averaging 7.5 MPa, indicating high sensitivity to test conditions. The DIC technique proved effective even under these critical conditions. In the ${\left[\pm 45\right]}_{3}$ laminate, greater strain was noted compared to the other configurations, and the DIC method demonstrated high precision in detecting strains, generating curves similar to those obtained by the analog extensometer.

The comparison between the theoretical and experimental results for the elastic modulus $E_{x}$ of the ${\left[\pm 45\right]}_{3}$ laminate revealed a 31.2% difference, indicating uncertainties in manufacturing and the absence of considerations about progressive damage in the theoretical analysis. The DIC technique with simplified equipment showed limits in detecting strain in more rigid conditions, with fibers aligned in the same direction as the load. However, for laminates with varied stacking sequences, a simplified DIC can be utilized. This study provides an important reference and basis for future research on the mechanical behavior of fiber-reinforced composites.

## Figures and Tables

**Table 1:** Table of sample sizing.

Stacking Sequence	Number of Samples	Dimensions of the Samples	Dimensions of the Plate
${\left[0\right]}_{3}$	10	^1^ 250 × 15 mm	315 × 230 mm
${\left[90\right]}_{4}$	10	^1^ 175 × 25 mm	250 × 310 mm
${\left[\pm 45\right]}_{3}$	10	^1^ 250 × 25 mm	275 × 280 mm

^1^ Dimensions in (length × width).

**Table 2:** Average of the properties obtained in the tests compared to the literature.

Mechanical Properties	DIC	Analog Extensometer	Mech-G [30]	Horlle [31]	Fiorelli [32]	Reis [33]
$E_{1}$ [MPa]	^1^ 34,459 ± 515 (1.5%)	^1^ 38,538 ± 1191 (3.1%)	44,816	20,600	29,187	23,930
$E_{2}$ [MPa]	^1^ 9581 ± 602 (6.3%)	^1^ 10,152 ± 757 (7.5%)	12,411	3800	-	7588
$\nu_{12}$	^1^ 0.29 ± 0.02 (6.9%)	-	0.28	0.27	0.30	0.30
$\nu_{21}$	-	^2^ 0.08 ± 0.01 (12.5%)	-	-	-	-
$G_{12}$ [MPa]	^1^ 2058 ± 24.4 (1.2%)	-	5515	-	-	2270
$\tau_{12}$ [MPa]	^1^ 28.2 ± 1.0 (3.5%)		68.0	-	-	31.4
$\sigma_{1}^{T}$ [MPa]	^1^ 567.4 ± 59.7 (10.5%)		1035	288.8	410	445.4
$\sigma_{2}^{T}$ [MPa]	^1^ 7.5 ± 0.2 (2.7%)		48	3.9	-	5.2

^1^ Mean ± standard deviation (coefficient of variation). ^2^ Poisson’s ratio 21 calculated from the reciprocity equation.

**Table 3:** Data obtained in the trials compared to the script.

	$E_{x}$		Relative Difference [%]	Relative Difference [%]
Analog Extensometer	DIC	Classical Laminated Plate Theory	Theory vs. Analog	Theory vs. DIC
^1^ 5208 ± 172 (3.3%)	^1^ 4945 ± 243 (4.9%)	7136	31.2	36.2

^1^ Mean ± standard deviation (coefficient of variation).

## Data Availability

The original contributions presented in the study are included in the article, further inquiries can be directed to the corresponding author.

## Abbreviations

The following abbreviations are used in this manuscript:

DIC	Digital Image Correlation
CNC	Computer numerical control
LED	Light-emitting diode
FPS	Frames per second
CFRP	Carbon fiber reinforced polymer
